# A phase 1, safety, tolerability, and pharmacokinetics study of bisnorcymserine, a highly selective inhibitor of butyrylcholinesterase

**DOI:** 10.1016/j.neurot.2026.e00918

**Published:** 2026-05-13

**Authors:** Iason Tzieras, Apostolos Manolopoulos, David Tweedie, Weiming Luo, Maria Maccecchini, Josephine M. Egan, Nigel H. Greig, Dimitrios Kapogiannis

**Affiliations:** a Laboratory of Clinical Investigation, National Institute on Aging, Intramural Research Program, Baltimore, MD, USA; b Translational Gerontology Branch, National Institute on Aging, Intramural Research Program, Baltimore, MD, USA; c Annovis Bio, Inc., Malvern, PA, USA

**Keywords:** Alzheimer’s disease, Cholinesterase inhibitor, Butyrylcholinesterase, Drug safety, Clinical trial

## Abstract

Cholinergic deficiency is a hallmark neurotransmitter abnormality in Alzheimer’s disease (AD) that has traditionally been addressed with cholinesterase inhibitors. In severe AD, butyrylcholinesterase (BuChE) becomes the dominant cholinesterase, suggesting a potential therapeutic target. (−)-N1,N8-bisnorcymserine tartrate (BNC) is a selective BuChE inhibitor designed to address this unmet need. We conducted a phase I, single-center, randomized, double-blind, placebo-controlled, ascending single oral dose clinical trial to evaluate the safety, tolerability, and pharmacokinetics of BNC in 30 healthy volunteers. There were no adverse events (AEs) grade 2 or above or any serious adverse events (SAEs). Most events were mild and self-limited, the most common being asymptomatic bradycardia and headache. The mean AUC_last_ (SD) was 120.98 h∗ng/mL (74.30) for the 40 mg dose, 148.20 h∗ng/mL (99.43) for the 80 mg dose, and 196.33 h∗ng/mL (91.74) for the 120 mg dose. Accordingly, median t_max_ (range) and mean C_max_ (SD) were 1.8 (1.0–5.0) hr and 13.94 (7.64) ng/mL for the 40 mg dose, 1.8 (1.5–5.0) hr and 18.54 (6.44) ng/mL for the 80 mg dose, and 2 (1.0–4.5) hr and 20.93 (5.00) ng/mL for the 120 mg dose. The mean half-life of BNC ranged from 5.5 to 7 h. BNC was safe and well tolerated when administered as a single oral dose of up to 120 mg. This first-in-human, phase I study permits further investigation of this drug as a potential symptomatic treatment for AD. ClinicalTrials.gov, NCT01747213.

## Introduction

The prevalence of Alzheimer’s disease (AD) continues to rapidly grow worldwide, with the number of individuals living with dementia projected to increase from 57 million today to 153 million by 2050 [1], while even more individuals have mild cognitive impairment (MCI) or preclinical AD [2]. In AD, new drug development is predominantly aimed toward preclinical, MCI and mild AD, when treatment may have the greatest potential impact on disease modification. Although anti-amyloid monoclonal antibodies have recently been approved for early AD [3], and symptomatic treatments, such as cholinesterase inhibitors and memantine, remain widely used [4], without the ability to halt disease progression, patients eventually advance to severe AD, where therapeutic options are limited. This highlights the need for novel treatment options to mitigate cognitive, behavioral, and functional symptoms in late AD.

The cholinergic system plays a key role in cognition, with acetylcholine being a critical signal transducer. Cholinergic neurons are extremely vulnerable to degeneration in AD, with cholinergic neuronal loss in the nucleus basalis of Meynert being an early event in the pathophysiology of AD that is tightly correlated to memory impairment. This evidence provided the basis for the cholinergic hypothesis of AD and led to the development of various agents seeking to boost cholinergic neurotransmission [5]. Cholinesterase (ChE) inhibitors are a result of this strategy, the first class of drugs to be granted approval for symptomatic treatment of AD. They remain widely used across mild, moderate and severe AD stages, despite more limited evidence of meaningful clinical benefit in severe AD, especially when used as a monotherapy [6]. The currently approved ChE inhibitors, donepezil, galantamine, and rivastigmine, chiefly target acetylcholinesterase (AChE), with the former two drugs being selective inhibitors and the latter being a non-selective inhibitor, since it also inhibits butyrylcholinesterase (BuChE) [5]. In the absence of AChE, butyrylcholinesterase inactivates the neurotransmitter acetylcholine (ACh) and is hence a potentially viable AD therapeutic target [[7], [8], [9]]. Whereas BuChE exerts about 20% of ChE activity in a normal brain [10], with a time-dependent cholinergic cell loss and an accompanying AChE decline occurring in AD [7,9], BuChE activity progressively increases with AD progression, by about 40% in the temporal cortex and by 65% in hippocampus, as shown by Perry et al. [11], and even becomes the dominant ChE subtype in certain brain regions, such as the entorhinal cortex, an important area for memory and a site of early pathology in AD [12].

Brain AChE is largely associated with the post-synaptic field of cholinergic neurons [13], while BuChE is predominantly found in glial cells [7,14]. Darvesh et al. [15] characterized distinct neuronal populations in the human amygdala that express BuChE rather than AChE, while in the human hippocampus, patterns of neuronal staining hinted at co-expression of BuChE and AChE. A distinguishing feature between AChE and BuChE is their concentration-dependent kinetics regarding ACh binding and cleavage. Whereas AChE-mediated ACh hydrolysis is highly efficient at low ACh concentrations, AChE becomes inhibited by excess substrate. The opposite occurs for BuChE, which is less efficient at low ACh concentrations, but is not inhibited by excess levels [16]. The local spatial relationship between neuronal AChE and largely glial BuChE suggests a supportive, compensatory role of BuChE in the healthy brain to co-regulate brain ACh by hydrolyzing only excessive levels [7,9,17]. With the loss of cholinergic neurons, depletion of ACh, and accompanying change in the ratio of BuChE to AChE with AD progression, BuChE likely leads to rapid hydrolysis of remaining ACh [17]. Therefore, developing a selective BuChE inhibitor to rescue the little remaining ACh may be beneficial.

To test this hypothesis, a series of BuChE selective inhibitors were designed on the backbone of the candidate AD drug (−)-phenserine tartrate by building out the *para*-position of the phenylcarbamoyl ring [18]. This resulted in several (−)-cymserine analogues, which were shown to elevate brain ACh levels, augment long-term potentiation, and improve the memory of aged rats, in the absence of any AChE inhibitory action or classical cholinergic adverse actions (central tremor, lacrimation) [19]. From these analogues, (−)-N1,N8-bisnorcymserine tartrate (BNC), a 110-fold selective BuChE inhibitor with a IC_50_ of <1 nM [[18], [19], [20]] was developed (Investigational New Drug (IND) application #109540).

In preclinical studies, BNC showed an extensive metabolism, converting to eight metabolites during incubations *in vitro* with suspensions of cryopreserved hepatocytes prepared from mice, rats, dogs and humans. The proposed metabolites were formed by various reactions, including possible demethylation, oxidation, reduction, and glucuronidation. The major metabolite observed across all four species was an oxidative product. All Phase 1 metabolites detected in human hepatocytes were also found in hepatocytes from at least one laboratory animal species. In humans, a significant minor metabolite emerged due to glucuronidation and was not present in other species, although mice and dogs produced a different glucuronidated and oxidative metabolite.

A first-in-human Phase I study of oral BNC was conducted as a single-center, randomized, double-blind, placebo-controlled, ascending single-dose safety, tolerability and pharmacokinetic study in healthy volunteers aged ≥55 years.

## Materials and Methods

### Study design

The doses tested were 20, 40, 80, and 120 mg, with each dose being tested separately in cohorts of 8 subjects. The original prespecified doses were 20, 40, 80, 120, 160, 270 and 380 mg. However, our study was terminated early, due to inability to enroll subjects for more than 1 year when evaluating the 120 mg dose level. Each subject was randomized 3:1 (BNC:placebo) to receive a single dose of the study medication (either BNC or placebo) and could only participate in a single cohort. Six subjects in each cohort received the active drug, while two received placebo, similar to previous phase I studies testing AChE inhibitors [21]. The exception was in the 120 mg cohort, where three subjects received BNC and three placebo. This design ensured the gradual accumulation of subjects who received placebo and allowed for balanced statistical comparisons by the time that cohorts receiving higher BNC doses were enrolled.

Randomization was performed by the NIA pharmacist using a computer-generated random sequence, which was enclosed in sequentially-numbered, opaque, identical, sealed envelopes. A Data Safety Monitoring Board evaluated the safety and tolerability of each dose before approving the enrollment of a new cohort receiving the next higher dose.

This clinical trial was performed according to the principles of the Declaration of Helsinki and was approved by the Institutional Review Board of the National Institutes of Health (NIH).

(NIH IRB #: 13AG0034). The study was registered at ClinicalTrials.gov (NCT01747213).

### Study participants

Participants were healthy volunteers aged ≥55 years recruited from the community around the Baltimore-Washington, DC area through advertising. Inclusion criteria included good general physical and mental health (as determined by medical history, baseline physical examination, vital signs, clinical laboratory tests, and electrocardiogram (EKG)), a body mass index (BMI) of 18.5–34.0 kg/m^2^ inclusive, a total body weight of >50 kg, and a Mini-Mental State Examination (MMSE) score higher than 27 at screening and at Visit 2-Day 1. Participants were excluded if they had any clinically significant concomitant medical or psychiatric disorders, including active asthma within the last 10 years or chronic obstructive pulmonary disease, clinically significant laboratory abnormalities, a history of significant allergy to any drug or systemic allergic disease, positive screening tests for common bloodborne pathogens and drug abuse and dependency, based on urine sample testing at screening and at admission to the NIA Clinical Unit on study Day 1. Common age-related disorders (such as hypertension, type 2 diabetes mellitus, dyslipidemia, or hypothyroidism) were permitted, as long as they were under good control by diet and/or medications. Other exclusion criteria included the use of any tobacco products in the past 3 months, consumption of alcohol within 48 h prior to Visit 2, consumption of dietary supplements, such as Ginkgo biloba, St. John’s wort, and ginseng, within two weeks before screening, and medications that may have posed a risk to the subject or produced overlapping side effects with BNC, for example other cholinesterase inhibitors (such as donepezil) or other drugs with anticholinergic side effects (such as pyridostigmine, tricyclic antidepressants, meclizine, or oxybutynin). Women of childbearing potential were required to have a negative urine pregnancy test (minimum sensitivity 25 IU/L or equivalent units of human chorionic gonadotropin) at screening and again prior to study drug administration, while both men capable of fathering a child and women of childbearing potential were required to use an adequate method of contraception to avoid conception throughout the study and for up to 30 days following drug administration. Lactating females were also excluded from this study.

Participants were free to withdraw from the study at any time. Additionally, the principal investigator could terminate a subject’s participation if this was deemed in the subject’s best interest, for example, in the case of an intercurrent illness or the need for an excluded medication.

The study was originally designed to enroll up to 48 subjects and evaluate BNC doses of up to 320 mg. However, due to the low rate of recruitment, the study was terminated early, after collecting complete data from 30 subjects who received doses up to 120 mg. A subject was determined to have completed participation if they received study medication (BNC or placebo) and underwent all protocol-specified evaluations.

### Study drug

The study drug, BNC tartrate, was provided in powder form from QR Pharma, Inc. (now Annovis Bio, Inc., Malvern, PA), while the NIA pharmacist performed the drug and placebo formulations. The appropriate dose of BNC tartrate was weighed and used to fill gel capsules without any additives, such as starch. Identical capsules filled with microcrystalline cellulose filler were used as placebo. Participants received a single oral dose of the study capsule (BNC or placebo) with approximately 8 oz. (240 mL) of water and remained in an upright (>45°) position for 2 h thereafter.

### Study procedures

The study was conducted at the National Institute on Aging (NIA) Clinical Unit at the grounds of MedStar Harbor Hospital in Baltimore, MD. The study protocol, informed consent form, and the conduct of the study were approved and overseen by the NIH IRB. All participants gave written informed consent prior to study enrollment.

The screening visit was performed on an outpatient basis and included a symptom review questionnaire, medical history and physical examination performed by a licensed practitioner, administration of the MMSE, screening laboratory tests, urine tests including screening for drugs of abuse, and EKG.

Within 21 days from screening, qualified consenting subjects arrived at the NIA Clinical Unit between 3:00–5:00 p.m. for an overnight stay. They filled out the symptom review questionnaire and had an EKG, safety laboratory tests and urinalysis with drug screen, and MMSE. Additionally, a urine pregnancy test was performed in women of childbearing potential. Participants had a regular diet dinner and an evening snack followed by fasting (except for water) from midnight until lunchtime on the following day. At about 7:00–8:00 a.m. the following day, an indwelling catheter or needle was inserted for facilitation of frequent blood draws. Subjects had a total of 19 blood samples for plasma pharmacokinetic (PK) measurements of drug levels. Venous blood (3 mL) was collected at Time 0 (prior to dosing) and at 0.25, 0.5, 1, 1.5, 2, 2.5, 3, 3.5, 4, 4.5, 5, 6, 8, 10, 12, and 16, 24 and 32 h after study medication administration. Orthostatic vital sign (blood pressure and heart rate) measurements, a resting 12-lead EKG, MMSE administration, and safety laboratory tests were obtained at 2.5 h (corresponding to the estimated time of peak drug concentration, t_max_, based on PK studies of BNC in dogs), and again at 6 and 12 h after study medication administration, whereas continuous cardiac monitoring was maintained from prior to drug administration until discharge. Following a second overnight stay, participants underwent blood draws at 24 and 32 h post-dose for plasma PK measurements, along with orthostatic vital signs and resting 12-lead EKGs. Within 1 h before the 32-h time point, urine and blood were collected for safety laboratory tests, and participants completed the symptom review questionnaire, and the MMSE. After discharge, participants were contacted by NIA clinical staff within 3 days to inquire about symptoms using the symptom review questionnaire. A follow-up visit occurred 7 ± 2 days after drug administration, during which the same assessments as during screening were performed. Participants with no adverse events (AEs) and normal laboratory values were then discharged from the study.

### Pharmacokinetic analyses

Plasma BNC measurements were processed from 3 mL blood samples obtained in dipotassium ethylenediamine tetraacetic acid (K2EDTA) tubes placed on wet ice and centrifuged within 10–15 min from withdrawal. The samples were stored in an ultralow −70 °C freezer until being shipped for analysis. The concentrations of BNC were measured using high-performance liquid chromatography (HPLC) with tandem mass spectrometry (MS/MS). The BNC concentration measurements to derive pharmacokinetics in the first cohort (20 mg) were performed by an independent contractor, SRI International (Menlo Park, USA). Quantification for the remaining cohorts (40, 80, and 120 mg) was performed by a different contractor, Resolian Pharma (Malvern, USA).

The LC-MS/MS data acquisition for samples was performed on a Shimadzu 30 series UPLC (Shimadzu Nexera X2) coupled with a Sciex API 5500+ mass spectrometer. Separation was performed on an Acquity UPLC BEH C18 column (1.7 m, 2.1 mm × 50 mm). Mobile phase A was 0.1% formic acid (FA, Optima HPLC grade, Thermo Fisher Scientific, Waltham, Massachusetts) in water, and mobile phase B was 0.1% FA in acetonitrile (ACN, HPLC grade, Thermo Fisher Scientific, Waltham, Massachusetts) at a flow rate of 0.6 mL/min. The internal standard was tolbutamide internal standard (TBA-IS, Toronto Research Chemical, Ontario, Canada). Prior to analysis, all samples were spiked with 1 M dichlorvos at a v/v ratio of 1:100 while still frozen. A 50.0 μL aliquot of each study sample was extracted with a protein precipitation method by adding 300 μL of internal standard solution (25.0 ng/mL of TBA-IS in ACN) in a 96-well sample plate. Following centrifugation at 3500 rpm for 10 min at room temperature, the supernatant was diluted 1:2 in HPLC water and mixed well prior to the LC-MS/MS analysis.

The pharmacokinetic parameters were determined by a non-compartmental model using Pharsight Phoenix WinNonlin® 6.3 software. Chromatograms were integrated using Analyst version 1.7.3 software. A weighted (1/x^2^, x = concentration) linear regression was used to generate the calibration curves for BNC. The concentrations of BNC were calculated using the peak area ratio of analyte to internal standard based on the standard curve. Time points were automatically selected by a “best fit” model for the terminal half-life (t_½_) estimation. The non-compartmental analysis (NCA) method of area under the curve (AUC) trapezoidal integration used was Linear Trapezoidal Linear Interpolation for AUC parameter estimation. The method of the first contractor had a lower limit of quantitation (LLOQ) of 0.05 ng/ml and the inter-batch precision across all concentrations and analytical runs ranged from 7.4% to 11.8%, and the inter-batch accuracy ranged from 99.6 % to 104.2%. For the second contractor, LLOQ was 1.00 ng/ml, inter-batch precision ranged from 5.9% to 8.9% and inter-batch accuracy from 90.7% to 108.7%.

### Statistical analyses

Statistical analyses were performed using RStudio statistical software (Boston, MA), version 2025.05.1 + 513 and GraphPad Software (San Diego, CA) Prism, version 10.0.2. Demographic data are presented using standard descriptive statistics. Baseline group differences were compared using Kruskal-Wallis (KW) nonparametric testing with post hoc Dunn’s test for multiple comparisons.

For analysis of quantitative variables, we fitted linear mixed models for repeated measures with group (20 mg, 40 mg, 80 mg, 120 mg, placebo), time, and group∗time interaction as fixed effects, individual participant IDs as random effect, and values at baseline as covariate. All quantitative variables were log-transformed due to their non-normal distributions. Safety data analysis included comparisons between dosage groups and placebo with respect to AEs, vital signs, laboratory test results, questionnaires on physical and psychological symptoms, and EKG findings. Additionally, vital signs at the time of maximum plasma drug concentration (t_max_) were compared among groups using Kruskal-Wallis nonparametric testing.

We performed additional exploratory analyses to examine the effects of important covariates on drug effects. We assessed main effects of age, BMI, sex, and race, and their interaction effects with drug concentration on safety outcomes. Model fit was evaluated using the Akaike information criterion (AIC) and the Bayesian information criterion (BIC); the model including group∗time interaction and baseline value was selected as the best fit based on lower AIC and BIC values.

PK parameters are summarized per cohort and include AUC_last_, C_max_, t_max_, and t_½_. Comparison of PK parameters among dosage groups was performed using Kruskal-Wallis nonparametric testing. To evaluate dose proportionality, subject-level PK parameters were analyzed using a power model on the log-transformed scale, with AUC_last_ and C_max_ modeled separately as a function of dose. Graphs of plasma concentration over time for individual subjects are also presented.

Safety analyses included data from all the participants who received at least one dose of the study treatment (BNC or placebo). PK parameters were analyzed in all participants who received a dose of BNC and had evaluable PK data. For all analyses, *p* values of <0.05 were considered statistically significant. No adjustments for multiple comparisons were applied, consistent with the exploratory nature of the study.

## Results

**Fig. 1:**
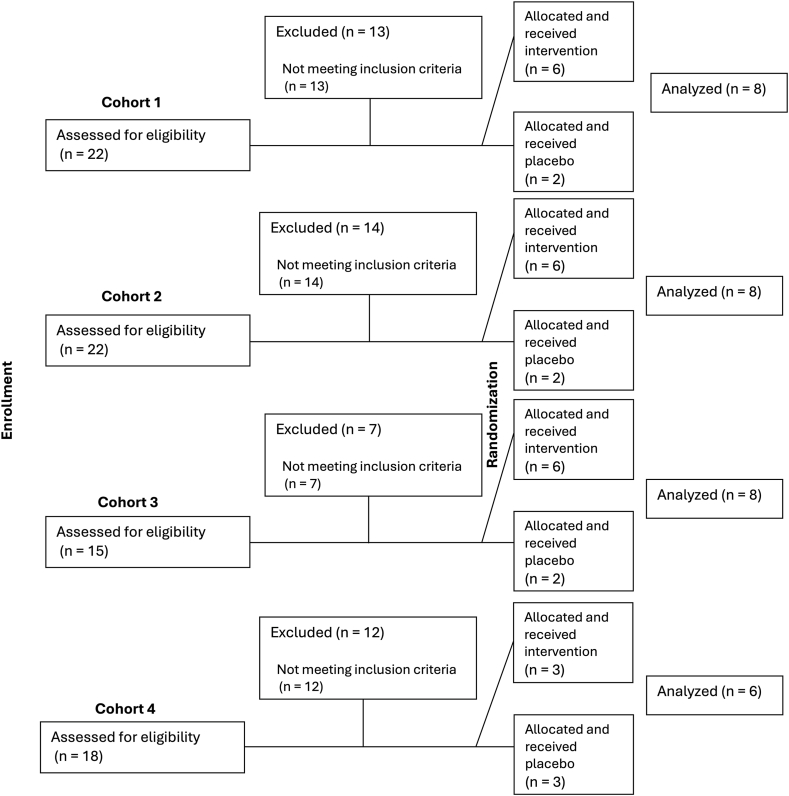
Study flowchart.

Between January 2013 and September 2018, seventy-seven participants were screened, of whom 47 were deemed ineligible due to abnormal laboratory values, EKG findings, or abnormal vital sign parameters. Thirty participants were included and randomized to receive BNC or placebo and completed all study procedures. They were assigned to cohorts receiving 20 mg, 40 mg, 80 mg, or 120 mg, each with a corresponding placebo group (Fig. 1). Sixteen (53%) participants were male and 14 (47%) female, with a mean age of 61 years (standard deviation (SD) 4.7) and a mean MMSE score of 29.3 (1.02) (Table 1). Four protocol deviations were reported to the IRB, all of which were minor and unlikely to have affected the analyses or conclusions of the study.

**Table 1:** Demographic and baseline characteristics.

Characteristic	Placebo *N*= 9	20 mg *N*= 6	40 mg *N*= 6	80 mg *N*= 6	120 mg *N*= 3	*P* value^b^
Sex – N (%)						0.43
Female	5 (56%)	1 (17%)	4 (67%)	2 (33%)	2 (67%)	
Male	4 (44%)	5 (83%)	2 (33%)	4 (67%)	1 (33%)	
Age – years	63.0 (5.4)	60.2 (2.5)	64.0 (4.9)	57.0 (2.3)	61.0 (5.6)	0.07
Weight – kg	78.1 (10.2)	85.7 (8.9)	70.8 (12.1)	77.3 (14.8)	85.1 (11.8)	0.29
BMI – kg m^−2^	27.4 (2.1)	28.0 (2.9)	24.7 (3.1)	24.1 (2.5)	27.9 (1.9)	0.048
Race^a^ – N (%)						0.58
HW	0 (0%)	0 (0%)	1 (17%)	0 (0%)	0 (0%)	
MORE	1 (11%)	0 (0%)	0 (0%)	0 (0%)	0 (0%)	
NHB	1 (11%)	3 (50%)	1 (17%)	3 (50%)	1 (33%)	
NHW	7 (78%)	3 (50%)	4 (67%)	3 (50%)	2 (67%)	
Ethnicity – N (%)						1
Hispanic	1 (11%)	0 (0%)	1 (17%)	0 (0%)	0 (0%)	
Non-Hispanic	8 (89%)	6 (100%)	5 (83%)	6 (100%)	3 (100%)	
MMSE	29.3 (1.0)	29.3 (1.2)	29.0 (0.9)	29.5 (1.2)	29.3 (1.1)	0.73

Values presented are means (standard deviation), unless otherwise stated.

BNC = (−)-N1,N8-bisnorcymserine; HW = Hispanic White; MORE = More than one race; NHB = Not Hispanic Black or African American; NHW = Not Hispanic White.

a Race was determined by the participants.

b *P* values are from the Kruskal–Wallis test.

### Safety and tolerability

The drug was well tolerated. A total of 19 AEs were reported, the most common being asymptomatic bradycardia and headache (Table 2). All events were mild and self-limited with some participants experiencing more than one AE. No AEs of grade ≥2 and no serious adverse events (SAEs) were observed that would define any tested BNC dose as the maximum tolerated dose. There was no association between higher BNC doses and incidence or severity of AEs.

**Table 2:** Summary of treatment-emergent adverse events by System Organ Class and Preferred Term.

System Organ Class	Preferred Term	Placebo *N*= 9	20 mg BNC *N*= 6	40 mg BNC *N*= 6	80 mg BNC *N*= 6	120 mg BNC *N*= 3	Total *N*= 30	Relatedness to study intervention
Cardiac disorders	**Bradycardia**	1	1	0	1	2	5	Possible
Cardiac disorders	**Arrhythmia**	0	0	0	1	0	1	Possible
Nervous system disorders	**Headache**	1	0	1	0	1	3	Possible
Nervous system disorders	**Dizziness**	0	0	0	1	0	1	Possible
Gastrointestinal disorders	**Nausea**	1	0	0	1	0	2	Possible
Investigations	**Blood triglycerides 003increased**	1	1	0	0	0	2	Unrelated
Skin and subcutaneous tissue disorders	**Pruritus**	0	0	0	1	0	1	Possible
General disorders and administration site conditions	**Infusion site edema**	0	0	0	1	0	1	Related^a^

Values represent the number of participants experiencing at least one occurrence of each adverse event. No participant experienced more than one event of the same type. Between-group comparisons were performed using Fisher’s exact test and were not statistically significant.

BNC = (−)-N1,N8-bisnorcymserine.

a Related to the study procedures (blood draws) rather than the pharmacologic action of the study drug.

Of note, in the 80 mg cohort, one participant developed mild scalp and facial pruritus approximately 2.5 h after drug dosing, coinciding with peak plasma drug concentration. The pruritus was intermittent and resolved spontaneously within an hour without recurrence. At around the same time, the participant experienced mild orthostatic dizziness. Approximately 17 h post-dose, the same individual exhibited three runs of ventricular tachycardia during sleep. The participant’s weight-adjusted dose was close to the cohort mean and these events were considered consistent with an exaggerated idiosyncratic response to cholinergic exposure.

**Fig. 2:**
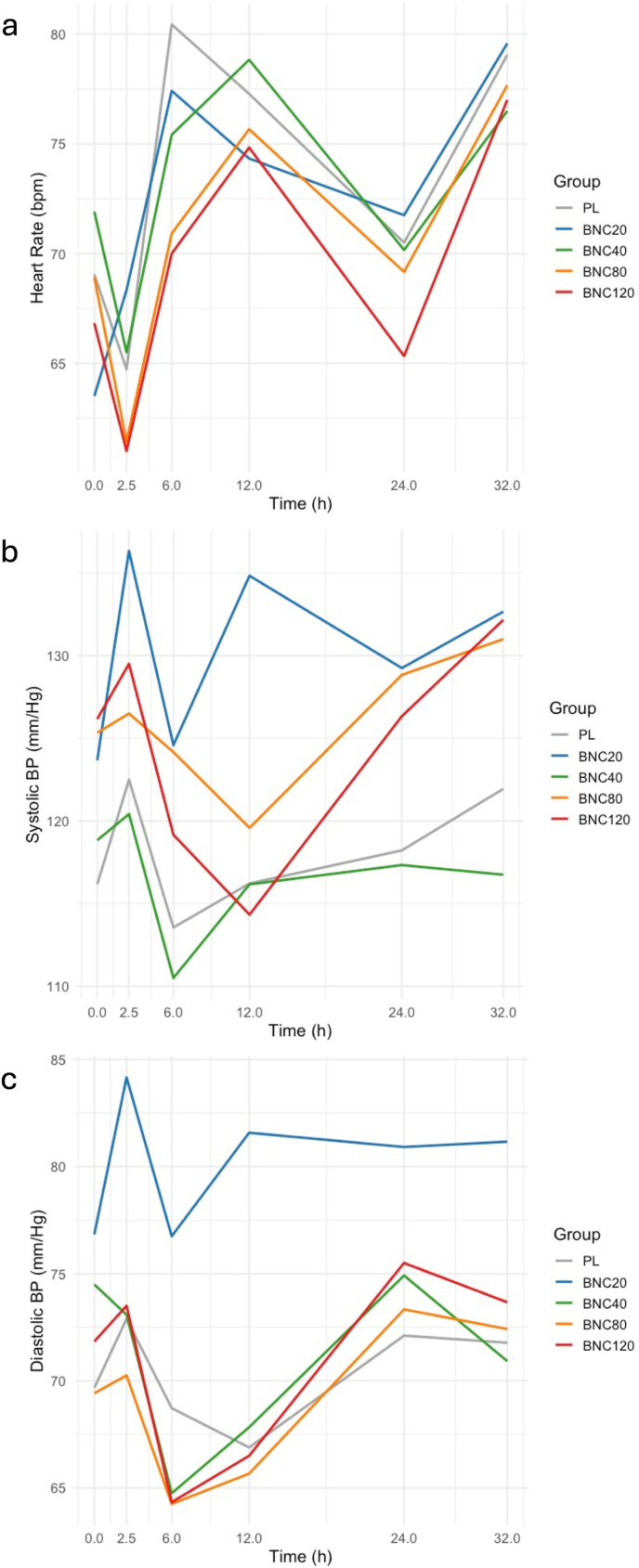
Heart Rate (a), Systolic Blood Pressure (b), and Diastolic Blood Pressure (c) by group over time. *No significant differences in heart rate were observed among treatment groups. The* 20mg BNC *group showed significantly higher systolic blood pressure compared with placebo (at 2.5, 6, 12 and 24 h after drug administration) and with the* 40mg *group both across and at each individual time point. The* 20mg *group also exhibited significantly higher diastolic blood pressure compared with placebo, the* 80mg*, and the* 40mg *group*, *both across and at each individual time point, and with the* 120mg *group (at 2.5, 6, 12 and 24 h after drug administration).*

**Fig. 3:**
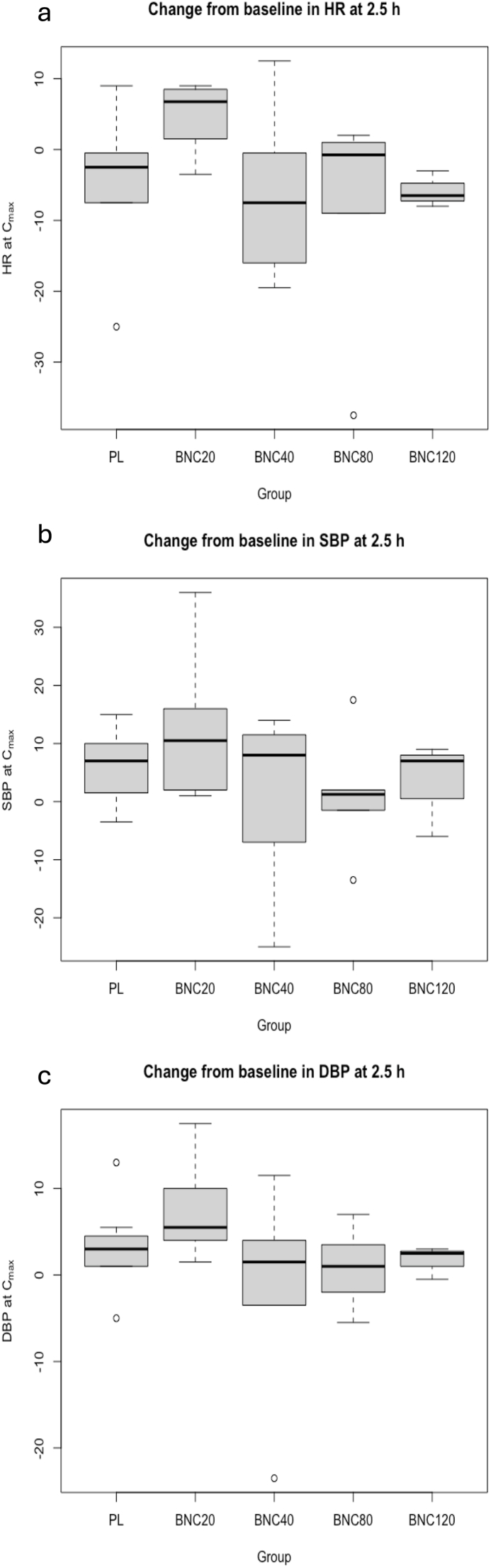
Change of Heart Rate (a), Systolic Blood Pressure (b), and Diastolic Blood Pressure (c) from baseline by group at average t_max_. *No significant differences in change from baseline in heart rate, systolic blood pressure or diastolic blood pressure were observed at average t*_*max*_*among the different groups.*

There were no significant differences in heart rate among treatment groups (Chi^2^_group∗time_= 4.76, *p*= 0.31; all pairwise comparisons across time points *p*> 0.05) (Supplementary Table 1). A statistically significant difference for systolic (Chi^2^_group∗time_= 3.59, *p*= 0.46) and diastolic blood pressure (Chi^2^_group∗time_= 2.49, *p*= 0.65) was observed, with the 20 mg group (lowest dose) showing higher values compared to other groups; this difference was present at baseline (prior to study drug administration) and persisted throughout subsequent time points (Fig. 2a–c), likely reflecting random variation (Supplementary Table 1). Specifically, for systolic blood pressure, the 20 mg group had higher values overall compared to the 40 mg group (difference in estimated marginal means [Δemmeans] 0.04, SE 0.01, *p*= 0.009) and to the placebo group at 2.5, 6, 12, and 24 h after drug administration (overall Δemmeans 0.02, SE 0.01, *p*= 0.06) (Supplementary Table 1). Similarly, for diastolic blood pressure, the 20 mg group exhibited higher values overall compared to the placebo group (Δemmeans 0.03, SE 0.01, *p*= 0.03), the 40 mg group (Δemmeans 0.05, SE 0.01, *p*= 0.001), the 80 mg group (Δemmeans 0.04, SE 0.01, *p*= 0.01), and the 120 mg group at 2.5, 6, 12, and 24 h after drug administration (all pairwise comparisons *p*< 0.05) (Supplementary Table 1). At the average time of peak plasma drug concentration (t_max_), no differences were observed among groups in the change from baseline of heart rate, SBP, or DBP (Fig. 3a–c). There were no between-group differences in PR, QT or corrected QT (QTc) intervals. Across time points, several minor between-group differences were observed in clinical laboratory measures, without clinical significance and likely the result of random variation (Supplementary Table 1).

### Pharmacokinetics

**Fig. 4:**
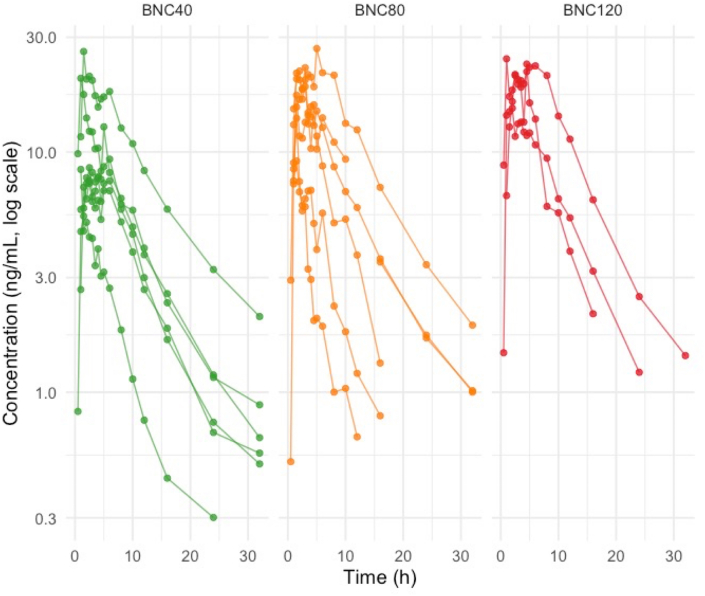
Plasma BNC concentration over time. Solid lines represent individual subjects.

**Fig. 5:**
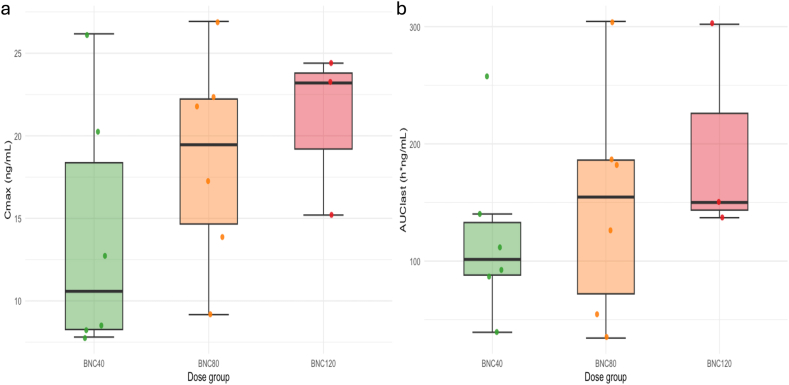
Exposure data, C_max_ (a) and AUC_last_ (b) by group. *No significant differences in C*_*max*_*or AUC*_*last*_*were observed among the different groups.*

We calculated standard PK parameters. In the 20 mg cohort, BNC plasma concentrations were below the assay LLOQ, except for one value just above the threshold; therefore, PK parameters for this group could not be determined and are not reported. No significant differences in PK parameters were observed among the remaining dosage groups (Fig. 4, Fig. 5). For the 40 mg, 80 mg, and 120 mg cohorts, mean maximum BNC plasma concentration (C_max_) were 13.94 ng/mL (SD 7.64), 18.54 ng/mL (6.44), and 20.93 ng/mL (5.00), respectively. Mean observed exposures (AUC_last_) were 120.98 h∗ng/mL (SD 74.30), 148.20 h∗ng/mL (99.43), and 196.33 h∗ng/mL (91.74), respectively. Median time to maximum concentration (t_max_) was approximately 2 h across all groups and the mean half-life (t_1/2_) ranged from 5.5 to 7 h (Table 3). Dose proportionality assessment could not support a linear relationship between dosage and exposure with a slope estimate of 0.468 (95% CI, −0.068 to 1.003) for C_max_ and 0.415 (95% CI, −0.456 to 1.286) for AUC_last_. There was substantial inter-subject variability with coefficient of variation (CV%) ranging from 23.9% to 54.8% for C_max_ and 46.7%–67.1% for AUC_last_ across dose groups (Table 3).

**Table 3:** Plasma pharmacokinetics of BNC.

Parameter	20 mg BNC *N*= 6	40 mg BNC *N*= 6	80 mg BNC *N*= 6	120 mg BNC *N*= 3	*P* value^d^
C_max_ – ng/mL^a^	BLQ	13.94 ± 7.64 (54.8%)	18.54 ± 6.44 (34.74%)	20.93 ± 5.00 (23.89%)	0.2420
t_max_ – hr^b^	NC	1.8 (1.0–5.0)	1.8 (1.5–5.0)	2.0 (1.0–4.5)	0.9070
t_½_ – hr^c^	NC	7.05 ± 0.99	5.60 ± 1.77	5.42 ± 0.99	0.1974
AUC_last_ – h∗ng/mL^a^	NC	120.98 ± 74.30 (61.41%)	148.20 ± 99.43 (67.09%)	196.33 ± 91.74 (46.72%)	0.4382
AUC_inf_ – h∗ng/mL^a^	NC	130 ± 82.56	157.70 ± 105.38	208.33 ± 92.51	0.3548
AUC Extr - %^c^	NC	6.3 ± 1.44	6.7 ± 1.99	6.36 ± 2.35	0.9858
CL/F – L/h^c^	NC	425.70 ± 281.73	853.42 ± 724.29	643.65 ± 228.87	0.4693

AUC_last_= area under the concentration–time curve from the time of dosing to the time of the last measurable concentration; AUC_inf_= area under the concentration–time curve from the time of dosing extrapolated to infinity; AUC Extr = percentage of AUC_inf_ obtained by extrapolation beyond the last measurable concentration; BLQ = below the lower limit of quantitation; BNC = (−)-N1,N8-bisnorcymserine; CL/F = apparent oral clearance; C_max_= maximum drug concentration observed in plasma after dosing; t_max_ = time for maximum concentration; t_1/2_= elimination half-life; NC = not calculable.

a Values presented are means ± standard deviation (coefficient of variation %).

b Values presented are medians (min–max).

c Values presented are means ± standard deviation.

d *P* values are from the Kruskal–Wallis test.

## Discussion

In this phase I, single-center, first-in-human study, we evaluated the safety, tolerability, and PK of ascending doses of BNC, a novel selective BuChE inhibitor, in healthy individuals aged ≥55 years. Overall, BNC was well tolerated across all dose levels, with no dose-limiting toxicities and no meaningful differences in PK parameters among doses.

Developing disease-modifying treatments for AD, which almost universally target patients with early-stage disease, has rightfully been a priority for researchers, due to their potential to alleviate the projected increasing incidence of the disease. However, patients with advanced disease have been somewhat neglected in the development of new treatment strategies. In brains that have undergone substantial degeneration, BuChE becomes the dominant cholinesterase subtype and its selective inhibition may present a viable possibility for symptomatic modification in later-stage disease [7,9,11,17,22]. Histochemical studies have revealed an altered distribution of AChE and BuChE in AD brain, with both being associated with Aβ plaques [22,23]. Moreover, in the vicinity of Aβ plaques, the biochemical properties of AChE and BuChE are altered compared to normal brain parenchyma [24]; including their optimal working pH, substrate affinities and inhibitor sensitivities [25]. BuChE may even be involved in Aβ plaque maturation and transformation from a benign to a pathological structure [24,25].

In this study, no SAEs or dose-limiting toxicities, including clinically significant abnormalities in vital signs or laboratory measures, were observed for any of the investigated doses. Side effects consistent with systemic cholinergic hyperactivity, such as bradycardia or headache, occurred but were mild and self-limiting, and comparable across dose groups. Overall, these events were consistent with the mechanism of action and the known class profile of cholinesterase inhibitors, and we did not observe any unexpected safety signals within the dose range evaluated. The PK analyses did not demonstrate any statistically significant differences in exposure (C_max_, AUC_last_) with increasing doses. This may reflect limited statistical power due to an overall small sample size and high inter-subject variability; however, it is possible that the tested doses reached a plateau in bioavailability. Dose proportionality assessment was inconclusive and did not support a clear linear relationship between dose and exposure. These results were consistent with BNC pharmacokinetics in canines, in which inter-animal variability was also high, with SD values ranging up to and sometimes over 50% of mean values. In addition, mean C_max_ increased in proportion to dose in both males and females for lower doses, but were less than proportional to dose (i.e., non-linear) in both males and females at higher doses. Preclinical data also suggest that bioavailability could have been affected by variable tissue distribution and extensive metabolism of BNC. Across treatment groups, the maximum observed plasma concentration of 20.93 ng/mL was reached at approximately 2 h in the 120 mg group, with the drug disappearing with a half-life that ranged from 5.5 to 7 h. These measures are comparable with those obtained in preclinical pharmacokinetic studies in canines, in which T_max_ varied from 1.17 to 3.33 h and the half-life ranged between 6.91 and 10.38 h.

Notably, BNC shares structural features with two other experimental drugs that are being developed for AD, posiphen (*Buntanetap*) and phenserine [[17], [18], [19],^26^]. The T_max_ of posiphen in healthy human volunteers was found to vary between 1.3 and 1.6 h, and its half-life between 4.0 and 5.5 h [26,27], while phenserine has an even shorter half-life [28], and hence its current clinical development employs a sustained release oral formulation. Posiphen and phenserine are primarily being metabolized via N-demethylation in their N1- and N8- positions [26,27] to generate pharmacologically active metabolites. BNC was developed as an already N-demethylated compound to short-circuit such metabolism and provide a more metabolically stable parent drug [17], which may account for the observed longer half-life of BNC than posiphen and phenserine across animal models and humans.

This study has several limitations. First, our small sample size provides limited power to extract confident conclusions about safety, vital sign changes over time and pharmacokinetic parameters, although this is typical for first-in-human phase 1 trials. This may be further compounded by the variability in PK parameters that may add variability in safety and physiologic parameters. Additionally, the single-dose design of the study does not provide any information about accumulation or longer-term safety outcomes from chronic exposure. Moreover, this study did not address the issue of target engagement. Lastly, substantial inter-subject variability in pharmacokinetic parameters may reflect individual differences in metabolism or tissue distribution.

Despite these limitations, this study also has some important strengths. It is the first study to clinically assess the safety, tolerability, and pharmacokinetic profile of a selective butyrylcholinesterase inhibitor at multiple dose levels. Importantly, there were no serious adverse events or dose-limiting toxicities within the investigated dose range, while the PK analysis provides an initial basis for dose selection for future studies. Future research should be conducted in larger cohorts with repeated-dose administration, pharmacodynamic assessments, and evaluation of target engagement. These steps will provide the necessary information to clarify the optimal dose range and further characterize both short- and longer-term safety.

In conclusion, BNC was safe and well tolerated in healthy participants aged 55 years and older. Targeting BuChE remains a promising approach for advanced AD. The favorable safety profile of BNC supports further clinical development.

## Author contributions

Conceptualization, D.K., N.H.G., M.M.; Methodology, D.K., N.H.G., M.M.; Validation, N.H.G., J.M.E., W.L., D.T.; Formal Analysis, I.T. and A.M.; Investigation, D.K., J.M.E.; Resources, N.H.G., M.M., J.M.E., W.L., D.T.; Data Curation, I.T.; Writing - Original Draft, I.T.; Writing - Review & Editing, D.K., N.H.G., M.M., J.M.E., W.L., D.T., A.M.; Visualization, I.T. and A.M.; Supervision, D.K., J.M.E.; Funding Acquisition, D.K., N.H.G.

All authors have reviewed and approved the final version of the manuscript.

## Data availability

De-identified individual will be deposited at the Mendeley Data Repository upon publication.

## Declaration of competing interest

The authors declare the following financial interests/personal relationships which may be considered as potential competing interests: Maria Maccecchini reports equipment, drugs, or supplies was provided by Annovis Bio Inc. Maria Maccecchini reports a relationship with Annovis Bio Inc that includes: employment and equity or stocks. If there are other authors, they declare that they have no known competing financial interests or personal relationships that could have appeared to influence the work reported in this paper.

## Appendix A Supplementary data

Multimedia component 1 Supplementary Table 1. Summary of measures.
